# Management of intracranial hemorrhage in adult patients on extracorporeal membrane oxygenation (ECMO): An observational cohort study

**DOI:** 10.1371/journal.pone.0190365

**Published:** 2017-12-21

**Authors:** Alexander Fletcher-Sandersjöö, Eric Peter Thelin, Jiri Bartek, Adrian Elmi-Terander, Mikael Broman, Bo-Michael Bellander

**Affiliations:** 1 Department of Neurosurgery, Karolinska University Hospital, Stockholm, Sweden; 2 Department of Clinical Neuroscience, Karolinska Institutet, Stockholm, Sweden; 3 Department of Clinical Neurosciences, Division of Neurosurgery, University of Cambridge, Cambridge Biomedical Campus, Cambridge, United Kingdom; 4 Department of Neurosurgery, Copenhagen University Hospital Rigshospitalet, Copenhagen, Denmark; 5 Department of Medicine, Karolinska Institutet, Stockholm, Sweden; 6 ECMO Center Karolinska, Department of Pediatric Perioperative Medicine and Intensive Care, Karolinska University Hospital, Stockholm, Sweden; 7 Department of Physiology and Pharmacology, Karolinska Institutet, Stockholm, Sweden; Universita degli Studi di Palermo, ITALY

## Abstract

**Background:**

Intracranial hemorrhage (ICH) is a common complication in adults treated with extracorporeal membrane oxygenation (ECMO). The aim of this study was to identify predictors of outcome and investigate intervention strategies following ICH development in ECMO-treated adult patients.

**Methods:**

We conducted a retrospective review of adult patients (≥18 years) who developed an ICH during ECMO treatment at the Karolinska University Hospital (Stockholm, Sweden) between September 2005 and May 2017. Outcome was assessed by 30-day mortality and Glasgow Outcome Scale (GOS) after 6 months. The statistical analysis was supplemented by a case series of patients who were surgically treated for an ICH.

**Results:**

Sixty-five patients developed an ICH during ECMO treatment. 30-day mortality was 74% (n = 48), and was significantly associated with low level of consciousness at ICH diagnosis (p = 0.036), presence of intraparenchymal hematoma (IPH) (p = 0.049), IPH volume (p = 0.002), presence of intraventricular hemorrhage (p = 0.001), subarachnoid hemorrhage Fisher grade (p<0.001), hydrocephalus (p<0.001), midline shift (p = 0.026) and absent basal cisterns (p<0.001). Among the 30-day survivors (n = 17), 63% (n = 10) had favorable neurological outcome (GOS 4–5) after six months. Five patients were surgically treated for their ICH, some with dire hemorrhagic consequences, however one patient made a complete recovery.

**Conclusions:**

ICH in adult ECMO patients is associated with a high mortality rate. Outcome predictors can help to identify patients where ICH treatment is indicated. Treating a patient with an ICH during ECMO represents an intricate balance between pro- and anticoagulatory demands. Furthermore, surgical treatment is associated with several risks but may be indicated in life-threatening lesions. Prospective studies are warranted.

## Introduction

Extracorporeal membrane oxygenation (ECMO) has become a mainstay of therapy in the treatment of severe reversible respiratory and/or circulatory failure, and is being used more frequently in adults [1–3]. There is, however, significant morbidity and mortality associated with the treatment itself [4]. Extensive hemorrhaging—a result of the systemic anticoagulation required to reduce circuit clotting—is one of the most common complications during ECMO [5]. Of these, intracranial hemorrhage (ICH) is probably the most devastating [6], with an in-hospital mortality of 70–92% in ECMO cohorts [7–10]. As the use of ECMO increases, so does the number of patients with ICH. Thus, improving the management of these patients is becoming increasingly crucial for ECMO centers worldwide. Although studies exist on predictors of ICH in ECMO patients [7–10], there are no published studies on intervention strategies or predictors of outcome following ICH development in ECMO-treated adult patients. While prospective, randomized studies are preferable, the presented results could be useful in supplementing the current literature and guiding future trial designs.

In this retrospective observational cohort study, we explored predictors of poor outcome, as well as potential management strategies, following ICH development in ECMO-treated adult patients.

## Materials and methods

### Patients

All adult (≥18 years) patients who developed an ICH during ECMO treatment at the Karolinska University Hospital, between September 2005 and May 2017, were included. Patients with the presence of an ICH on admission were excluded. Medical records, including clinical notes, laboratory analysis, monitoring reports and brain imaging data were retrospectively collected from digital hospital charts.

### Variables

The following data were collected for all patients upon ECMO initiation: indication for ECMO treatment, age, gender, Charlson comorbidity index (a scoring system that predict one-year mortality based on a patients’ comorbidities [11]) and pre-admission antithrombotic therapy (defined in our study as antiplatelet or anticoagulation therapy at the time of hospital admission).

The following data were collected for all patients at the time of ICH diagnosis: venoarterial (VA) or venovenous (VV) ECMO-mode, pre-diagnostic neurological symptom(s) (symptoms present prior to the performance of the diagnostic computerized tomography (CT) scan), ICH classification (intraparenchymal hemorrhage (IPH) (including hemorrhage volume and location), subdural hemorrhage (SDH), subarachnoid hemorrhage (SAH) (including Fisher grade [12]), intraventricular hemorrhage (IVH) (including LeRoux grade [13])), secondary ICH complications (ischemic stroke, hydrocephalus, midline shift and absence of basal cisterns) and level of consciousness (assessed using the Reaction Level Scale (RLS-85) [14], which is a Glasgow Coma Scale (GCS)-based combined stepwise scale that is used in Sweden, where it has shown better association with outcome than GCS [15]). IPH hemorrhage volume was calculated by multiplying the length × width × height and dividing by two [16].

The following data were collected for all patients after decannulation or death: 30-day mortality and the ICH intervention(s) used. The intervention methods were categorized into:

1. Hemostatic intervention: Withdrawal of the heparin infusion and/or admission of anti-fibrinolytics, heparin antagonists, platelets or platelet-stimulating agents.
2. Unmonitored intracranial pressure (ICP)-intervention: Hyperosmolar therapy, heavy sedation, hyperventilation and/or controlled hypothermia performed without invasive ICP monitoring.
3. Surgical intervention: Hematoma evacuation and/or external ventricular drain (EVD) placement.
4. Decannulation: Weaning off ECMO to further facilitate ICH treatment.
5. Withdrawal of life-sustaining treatment: Withdrawal of ECMO treatment and other life-sustaining measures deemed futile.

Furthermore, for patients where ECMO treatment was continued after ICH diagnosis, we calculated the mean activated clotting time (ACT), activated partial thromboplastin time (APTT), platelet count, fibrinogen concentration, international normalized ratio (INR), mean arterial pressure (MAP) and venous partial pressure of carbon dioxide (P_v_CO_2_) from ICH diagnosis to decannulation/death. For patients who survived past 30 days, the Glasgow Outcome Scale (GOS) [17] was used to assess outcome after 6 months. In patients who did not survive, we noted whether the ICH was the probable cause of death (defined either as death by neurologic criteria or following ECMO treatment withdrawal as a result of the ICH diagnosis).

### Patient management during ECMO treatment

Patients with potentially reversible acute respiratory and/or cardiac failure were considered for ECMO treatment. Normally, treatment was commenced at the referring hospital and the patient was then transferred to the ECMO intensive care unit (ICU) [18,19]. Anticoagulation was achieved by continuous intravenous infusion of unfractionated heparin targeting an APTT of 1.5 to 2 times the mean normal value, which was assessed three times daily. Within two to three days following arrival at the ICU, a tracheostomy was performed, after which sedation was reduced with the goal of keeping the patient awake when possible. The bedside nurse, as well as the physician in charge of the patient, continuously monitored the central nervous system through neurological examinations. This included response to verbal directives or pain, brainstem reflexes, eye opening and pupil examinations. When a neurological event occurred (e.g. seizure, pupillary abnormalities, confusion or decreased levels of consciousness) a cranial CT scan was performed accordingly. Additional cranial CT scans were also performed whenever the patient was referred for a thoracic or abdominal CT scan, even in the absence of a neurological indication.

### Statistical analysis

For descriptive purposes, continuous data are presented as medians (interquartile range) and categorical data as numbers (proportion). Mann-Whitney U test was used to compare continuous variables between survivors and non-survivors. For categorical variables, Chi-square test (pair-wise where applicable) or Fisher’s exact test were used depending on sample size distribution. Many of the parameters of clinical importance that were included shared a strong natural covariance, making it difficult to adequately interpret the results from uni- and multivariable regression analyses, thus these were not included in the study. The statistical significance level was set to p<0.05. The statistical program R (version 3.4.0) was used, utilizing the interface R-studio (version 1.0.143). The raw data is available as a supplementary file (S1 Dataset).

### Ethical considerations

The study was approved by the Regional Ethical Review Board in Stockholm, Sweden (Dnr: 2016/1498-31/4) who, in accordance with Swedish Law, waived the need for informed consent. Clinical data is stored in the patient’ s hospital charts, which are biometrically protected and stored in hospital servers. The extracted data were analyzed anonymously, with results presented on a group level, making it impossible to identify individual patients.

## Results

During the study period, 351 adult patients were admitted for treatment at our ECMO center. Of these, 69 (20%) were diagnosed with an ICH during ECMO treatment. Four patients were excluded due to the presence of an ICH at admission. Consequently, 65 patients were included in the study. The distribution of the Charlson comorbidity index was: 0 (n = 43), 1 (n = 12), 2 (n = 7), 3 (n = 2) and 6 (n = 1), indicating that the prevalence of comorbidities was low. There was no significant difference in Charlson comorbidity index, gender, age, ECMO mode or indication between the patients that were alive and deceased after 30 days (Table 1).

**Table 1:** Demographics, pre–admission morbidity, ECMO indication, ECMO mode and outcome.

Variable	Entire cohort (n = 65)	Alive after 30 days (n = 17)	Deceased within 30 days (n = 48)	p-value
Age (years)	51 (41–61)	47 (34.5–62)	52.5 (40–61)	0.351
Male gender	42 (65%)	8 (47%)	34 (71%)	0.143
Pre-admission antithrombotic therapy	8 (12%)	0 (0%)	8 (17%)	N/A
Charlson comorbidity index	0 (0–1)	0 (0–1)	0 (0–1)	0.650
ECMO indication				0.172
Pulmonary	52 (80%)	11 (65%)	41 (85%)	
Cardiac	7 (11%)	4 (24%)	3 (6%)	
ECPR	6 (9%)	2 (12%)	4 (8%)	
ECMO mode ^a^				0.093
Venovenous	23 (35%)	5 (29%)	18 (38%)	
Venoarterial	42 (65%)	12 (71%)	30 (63%)	
ICH = cause of death	39 (60%)	–	39 (81%)	N/A
6 month GOS 4–5	–	10 (59%)	–	N/A

Abbreviations: ECMO = Extracorporeal membrane oxygenation, ECPR = Extracorporeal cardiopulmonary resuscitation, ICH = Intracranial hemorrhage, GOS = Glasgow Outcome Scale, N/A = Not available

^a^ ECMO mode at ICH diagnosis

Values are expressed as median (interquartile range) or numbers (proportion). Pulmonary indications included pneumonia, sepsis, respiratory failure, ARDS and drowning. Cardiac indications included cardiogenic shock and pulmonary embolism.

### ICH classification and patient outcome

A combination of different forms of ICH was common. Out of the entire 65-patient cohort, 74% (n = 48) exhibited IPH, 54% (n = 35) SAH, 38% (n = 25) IVH and 9% (n = 6) SDH. Seventy-two percent (n = 47) of patients presented with neurological symptoms before the diagnostic cranial CT scan was performed, while in the remainder no apparent neurological indication was present at ICH diagnosis (Table 2). The 30-day mortality for the entire cohort was 74% (n = 48), with 81% (n = 39) of these deaths attributed to the ICH, clearly showing its devastating impact in these patients. Out of the 17 survivors, 59% (n = 10) showed a favorable neurological outcome (GOS 4–5) after six months.

**Table 2:** ICH presentation and characteristics.

Variable	Entire cohort (n = 65)	Alive after 30 days (n = 17)	Deceased within 30 days (n = 48)	p-value
**ICH presentation**				
RLS–85	8 (3.25–8) (27 missing, 42%)	4.5 (1.5–7.75) (9 missing, 53%)	8 (3.75–8) (18 missing, 38%)	**0.036**
Pre–diagnostic symptom ^a^	47 (72%)	10 (59%)	37 (77%)	0.258
**ICH characteristics** ^b^				
IPH	48 (74%)	9 (53%)	39 (81%)	**0.049**
Volume (mL) ^c^	28.88 (6.18–63.76)	2.40 (1.00–8.34)	37.96 (8.00–80.12)	**0.002**
Supratentorial	44 (68%)	9 (53%)	35 (73%)	0.738
Infratentorial	9 (14%)	1 (6%)	8 (17%)	0.859
SAH	35 (54%)	7 (41%)	28 (58%)	0.349
Fisher grade	3 (2–4)	2 (2–2)	4 (3–4)	**<0.001**
SDH	6 (9%)	3 (18%)	3 (6%)	0.379
IVH	25 (38%)	1 (6%)	24 (50%)	**0.001**
LeRoux grade	6 (4–12)	2 (N/A)	7.50 (4–12)	0.183
Hydrocephalus	31 (48%)	1 (6%)	30 (63%)	**<0.001**
Midline shift (mm)	0 (0–9)	0 (0–0)	0 (0–11)	**0.026**
Absent basal cisterns	36 (55%)	1 (6%)	35 (73%)	**<0.001**
Ischemic stroke	25 (38%)	4 (24%)	21 (44%)	0.161

Abbreviations: ICH = Intracranial hemorrhage, RLS-85 = Reaction Level Scale, IPH = Intraparenchymal hemorrhage, SAH = Subarachnoid hemorrhage, SDH = Subdural hemorrhage, IVH = Intraventricular hemorrhage

^a^ Symptoms included mydriasis, seizures, confusion, decreased levels of consciousness and abnormal breathing patterns

^b^ If several scans were performed, the “worst” CT scan was used

^c^ Calculated by multiplying the length x width x height of the hemorrhage and dividing by two

Values are expressed as median (interquartile range) or numbers (proportion). Bold text in the p-value column indicates a statistically significant correlation (p<0.05).

### Predictors of mortality

All eight patients with pre-admission antithrombotic therapy passed away within 30 days of ICH diagnosis (Table 1). Moreover, 30-day mortality was significantly associated with a low level of consciousness at ICH diagnosis (p = 0.036), presence of IPH (p = 0.049), IPH volume (p = 0.002), presence of IVH (p = 0.001), SAH Fisher grade (p<0.001), hydrocephalus (p<0.001), midline shift (p = 0.026) and absent basal cisterns (p<0.001) (Table 2).

### ICH management

**Fig 1:**
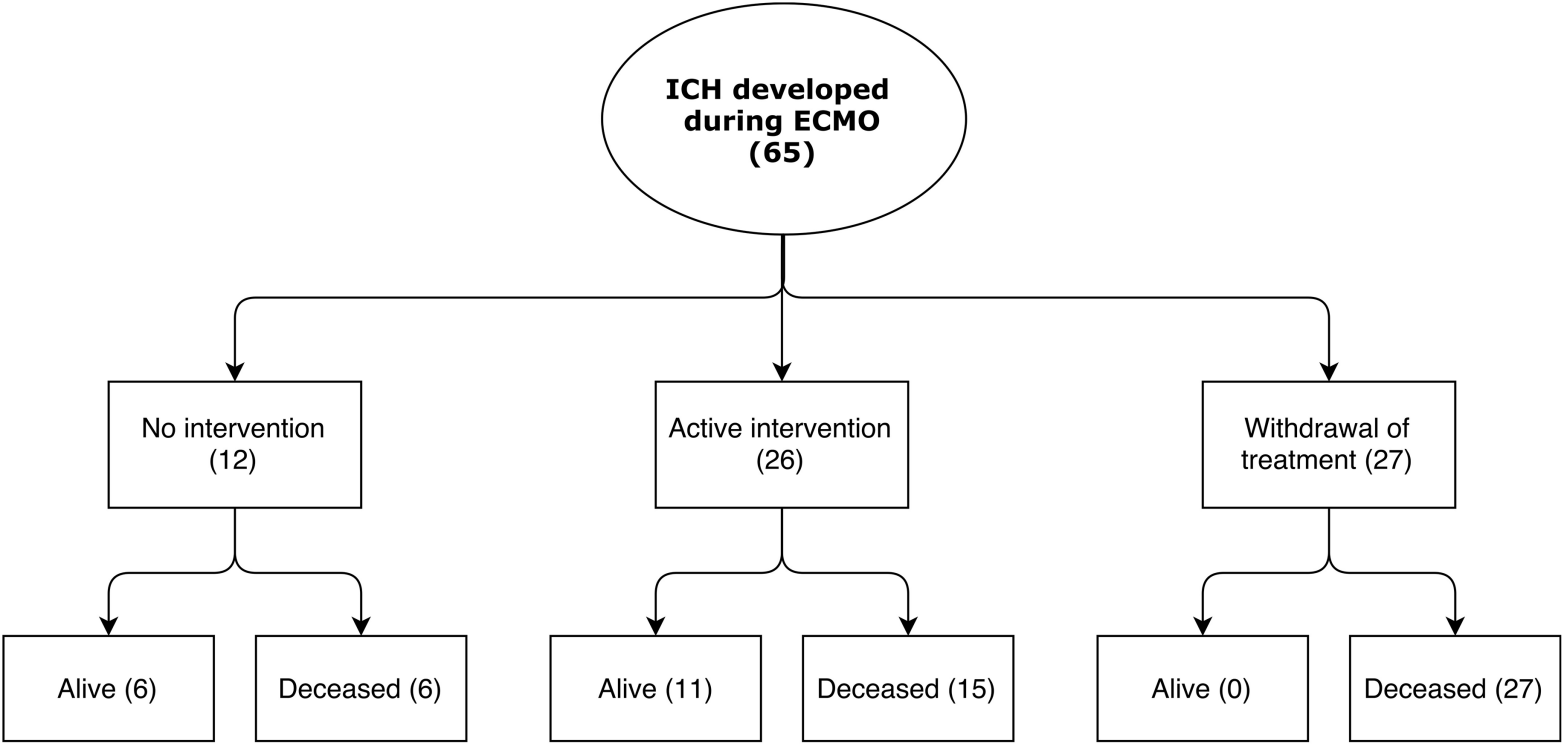
Patient management and outcome within 30 days of ICH diagnosis. Abbreviations: ICH = Intracranial hemorrhage; ECMO = Extracorporeal membrane oxygenation.

Following ICH diagnosis, in 42% (n = 27) of patients a decision was made to withdraw life-sustaining ECMO therapy. In contrast, the ICH was deemed to be of no clinical significance in 18% (n = 12) of patients, and consequently no active intervention was undertaken. Among these patients, there were no deaths attributed to the ICH. The remaining 40% (n = 26) of patients were subjects for treatment: 29% (n = 19) underwent hemostatic intervention, 22% (n = 14) underwent unmonitored ICP-intervention, 8% (n = 5) underwent surgical intervention, and 14% (n = 9) were uneventfully decannulated from ECMO (Table 3) (Fig 1). In the patients who continued on ECMO support, mean MAP, ACT, APTT, INR, platelet count, fibrinogen concentration and PvCO_2_, between ICH diagnosis and decannulation/death, did not predict mortality (S1 Table).

**Table 3:** ICH intervention and outcome.

Variable	Entire cohort (n = 65)	Alive after 30 days (n = 17)	Deceased within 30 days (n = 48)
No intervention	12 (18%)	6 (35%)	6 (13%)
Active intervention	26 (40%)	11 (65%)	15 (31%)
Surgical intervention	5 (8%)	2 (12%)	3 (6%)
Unmonitored ICP-intervention	14 (22%)	3 (18%)	11 (23%)
Hemostatic intervention	19 (29%)	7 (41%)	12 (25%)
Decannulation	9 (14%)	5 (29%)	4 (8%)
Withdrawal of life-sustaining treatment	27 (42%)	0 (0%)	27 (56%)

Abbreviations: ICH = Intracranial hemorrhage, ICP = Intracranial pressure. Values are expressed as numbers (proportion). Surgical intervention included hematoma evacuation and external ventricular drain placement. Unmonitored ICP-intervention included hyperosmolar therapy, heavy sedation, hyperventilation and controlled hypothermia performed without invasive ICP monitoring. Hemostatic intervention included withdrawal of the heparin infusion and admission of anti-fibrinolytics, heparin antagonists, platelets or platelet-stimulating agents. Decannulation was defined as weaning off ECMO to further facilitate ICH treatment. Withdrawal of life-sustaining treatment was defined as withdrawal of ECMO treatment and other life-sustaining measures deemed futile.

### Case series: Surgical intervention

While surgical treatment may be indicated when an ICH occurs during ECMO treatment, the associated anticoagulation presents a considerable risk factor. In literature, we have identified three cases of surgical intervention in patients with ICH during ECMO treatment—all of them IPHs treated with hematoma evacuation. In two of these cases, the patients passed away following peri-operative bleeding complications [20,21]. The third case did not experience any major peri-operative bleeding, but had regained only partial neurological function (GCS 11) after six months [22].

In the following section, we review the cases of five patients who were surgically treated for an ICH developed during ECMO treatment at our center.

#### Case 1

**Fig 2:**
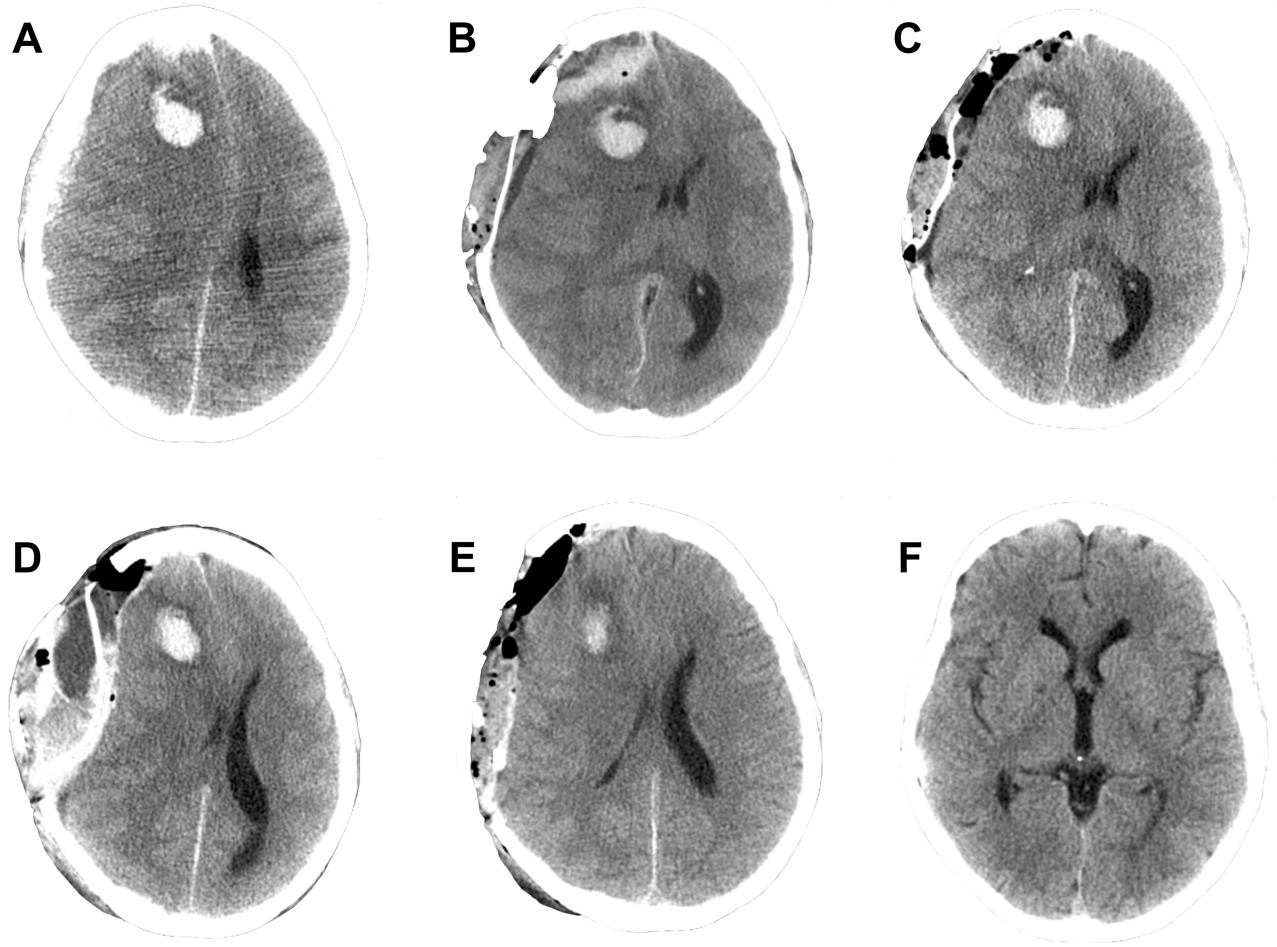
Case 1 cranial computerized tomography (CT) scans. A) CT scan following ICP-placement showing a frontal intraparenchymal hemorrhage (IPH), subdural hemorrhage (SDH) and midline shift. B) Post-operative CT scan following the first hematoma evacuation showing the development of a subgaleal hemorrhage, SDH recurrence and unchanged mass-effect as compared to pre-operative conditions. C) Post-operative CT scan following the second hematoma evacuation revealing improved conditions with no rebleeding, decreased midline shift and minor increase in IPH volume. D) CT scan the day after the second hematoma evacuation showing development of a subgaleal hemorrhage, SDH recurrence, epidural hemorrhage and increased midline shift. E) Post-operative CT scan following the third hematoma evacuation showing no rebleeding and decreased midline shift. F) CT scan one year after decannulation showing complete resorption of hemorrhages and no sign of secondary brain injury, as well as the bone flap replaced. Abbreviations: CT = Computerized tomography; IPH = Intraparenchymal hemorrhage, SDH = Subdural hemorrhage.

A 20-year-old woman, with a history of IgG deficiency, presented at her local outpatient clinic with shortness of breath and was prescribed antibiotics. One week later the patient was admitted to her local hospital with signs indicative of septic shock, and shortly thereafter she went into cardiac arrest and cardiopulmonary resuscitation (CPR) was initiated. Extracorporeal cardiopulmonary resuscitation (ECPR) was commenced using VA ECMO. Shortly after cannulation the patient developed bilateral mydriasis but the cranial CT scan did not show any apparent ischemia or intracranial mass lesions. To prevent development of cerebral edema, the patient was managed with hyperosmolar therapy, hyperventilation, hypothermia and heavy sedation (Pentobarbital coma monitored with burst suppression on continuous electroencephalography (EEG)). Despite this, a CT scan performed on ECMO day (ED) 6 revealed increased cerebral edema, and the patient underwent ICP catheter (Codman^®^) placement. No significant bleeding events occurred during the procedure, however, post-operatively her right-sided mydriasis increased. A cranial CT scan revealed a frontal IPH (6.75 ml, largest diameter 27 mm) and a SDH with 14 mm midline shift (Fig 2A). The ongoing heparin infusion was withdrawn, and 30 minutes later an emergency SDH hematoma evacuation was performed. The pre-operative coagulation parameters were: platelet count 69 x10^9^/mL, APTT 55 seconds, ACT 204 seconds and INR 1.0. Intra-operatively, no significant bleeding events occurred. However, the post-operative cranial CT scan revealed a subgaleal hemorrhage and an SDH recurrence with 14 mm midline-shift (Fig 2B). A new hematoma evacuation was performed without any significant intra-operative bleeding. A post-operative CT scan showed a decrease in the midline shift without any signs of hematoma recurrence (Fig 2C). However, the following morning her ICP increased drastically, and a new CT scan revealed a SDH and 20 mm midline shift, as well as epidural and subgaleal bleeding with impending uncal herniation (Fig 2D). Again, emergency hematoma evacuation was performed. Following this, the pupils regained their normal size and the CT scan showed a decreased midline shift without any signs of hematoma recurrence (Fig 2E). The ECMO treatment continued without heparin while prophylactic Levetirecetam was administered to prevent epileptic seizures. On ED 11 rewarming was successfully initiated, and the patient was decannulated from ECMO. At this stage, the patients had been without the heparin infusion for five days without any sign of complications. The patient was transferred to the neurosurgical ICU department, where extubation was successfully performed. After this the patient was transferred to a neurological care unit for rehabilitation. Within a year the patient had made a full recovery, with no neurological sequelae or physiological deficits, and had returned to work.

#### Case 2

**Fig 3:**
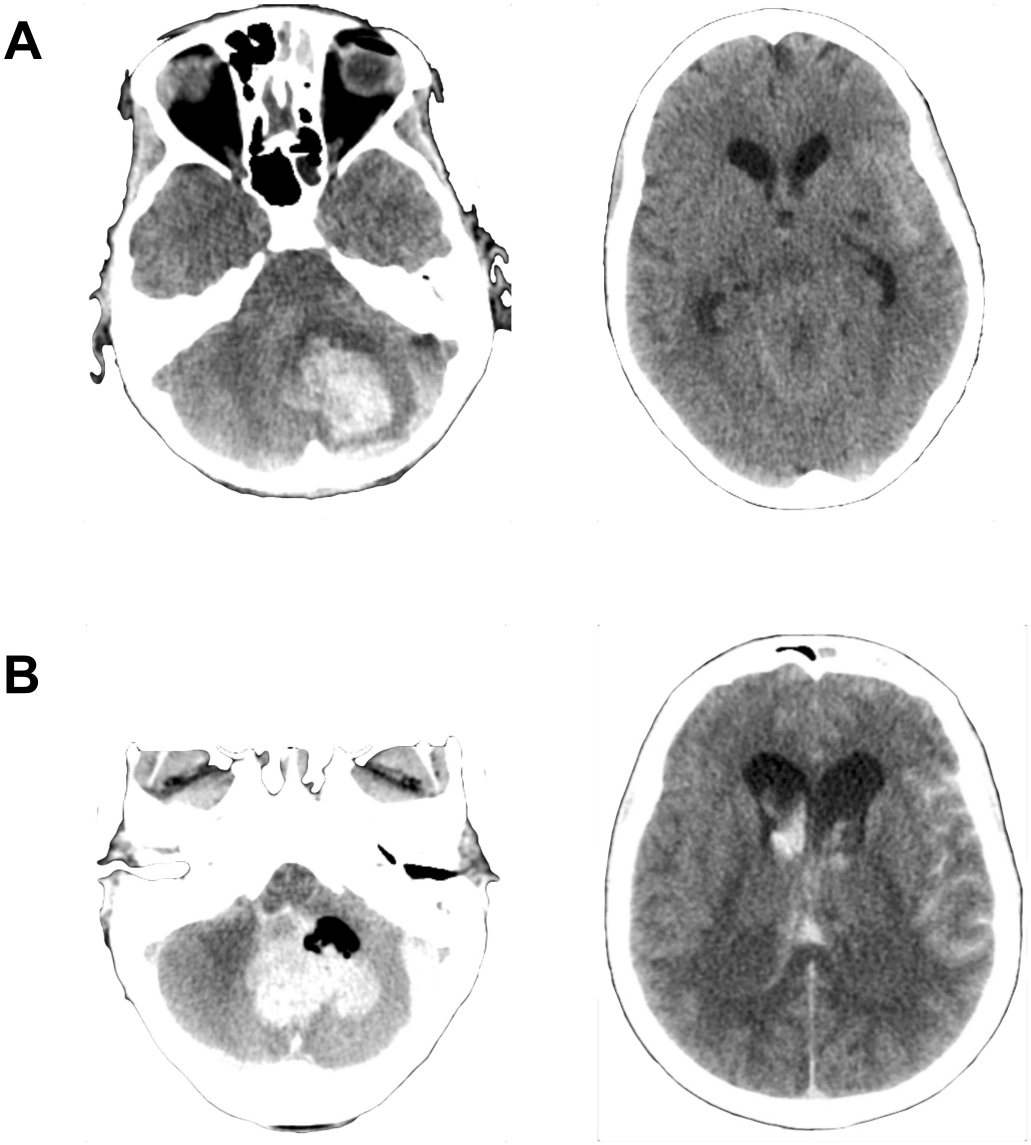
Case 2 cranial computerized tomography (CT) scans. A) Pre-operative CT scan showing cerebellar intraparenchymal hemorrhage (IPH), subarachnoid hemorrhage (SAH) and absent basal cisterns. B) Post-operative CT scan revealing increased IPH volume, progress of hydrocephalus, SAH and intraventricular hemorrhage (IVH) as well as tonsillar herniation. Abbreviations: CT = Computerized tomography; IPH = Intraparenchymal hemorrhage, SAH = Subarachnoid hemorrhage, IVH = Intraventricular hemorrhage.

A 35-year-old woman, with a history of depression, overdosed on a combination of antidepressants and alcohol, rendering her unconscious and resulting intubation and mechanical ventilation. Unfortunately, a later extubation attempt resulted in aspiration and severe respiratory failure to a degree that ECMO treatment was advocated, and the patient was started on VV ECMO. During the first week of ECMO, the patient developed progressive right ventricular heart failure (RVF), requiring conversion to VA ECMO. During the following weeks, the patient regained native lung function, but also experienced upper gastrointestinal bleeds requiring replacement of fibrinogen and platelets to prevent coagulopathy. On ED 32, the patient developed right-sided mydriasis and hypertension. A CT scan revealed a cerebellar IPH (23 mL, largest diameter 40 mm) with SAH (Fisher grade 4), IVH (Le Roux grade 4) and absent basal cisterns (Fig 3A). No bleeding source was found on a cerebral angiography. The heparin infusion was withdrawn, and protamine sulfate, fibrinogen, platelets and tranexamic acid was administered to reverse anticoagulation. Half an hour later an emergency cerebellar hematoma evacuation was performed. The pre-operative coagulation parameters were: platelet count 87, APTT 50, ACT 200, INR 1.0. Intra-operatively, opening of the dura resulted in uncontrollable hemorrhaging despite extensive use of local hemostatic agents and transfusions of fibrinogen, protamine sulfate, plasma, platelets, eptacog alfa (NovoSeven^®^, AryoSeven^®^) and cryoprecipitate. Due to the poor prognosis, an EVD was not placed. The post-operative CT scan showed increased IPH hematoma volume (41 ml) as well as progress of the hydrocephalus, IVH (LeRoux grade 12), SAH (Fisher grade 4) and tonsillar herniation (Fig 3B). Further ECMO treatment was deemed futile and, consequently, treatment was withdrawn and the patient passed away.

#### Case 3

A 68-year-old woman, with a history of hypertension, was admitted to her local hospital after three days of dyspnea. She was diagnosed with pneumonia with respiratory failure and was therefore intubated and administered intravenous antibiotics. Despite this, respiratory function continued to decline and ECMO treatment was advocated, and the patient was started on VV ECMO. On ED 1, RVF developed and the patient was converted to VA ECMO. On ED 6, after days of low platelet count despite transfusions, the patient developed right-sided mydriasis and decreased level of consciousness. A CT scan revealed a SAH (Fisher grade 4) with IVH (Le Roux grade 4) as well as hydrocephalus and absent basal cisterns. A cerebral angiography could not detect any bleeding source. Heparin was withdrawn, and four hours later an emergency EVD placement was performed. The pre-operative coagulation parameters were: platelet count 118, APTT 33, ACT 153 and INR 1.1. Intra-operatively, no significant bleeding events occurred. Post-operatively, a decision was made to keep the ECMO system running without heparin and to administer platelets. ICP was managed with hyperosmolar therapy and hyperventilation. Despite these measures, the patient did not regain consciousness. The following day the patient experienced a cardiac arrest and passed away.

#### Case 4

A 47-year-old woman, with a history of idiopathic nephrosis, hypertension and diabetes, was admitted to her local hospital after seven days of dyspnea, fever and myalgia. Intravenous antibiotics were administered for a suspected bacterial upper respiratory tract infection, and due to the severe dyspnea the patient was intubated. Despite this, respiratory function continued to decline, and ECMO treatment was advocated. The patient was cannulated for VV ECMO and transported to the ECMO ICU, where H1N1-influensa was diagnosed and appropriate treatment started. On ED 9 the patient developed left-sided mydriasis and level of consciousness decreased. A CT scan revealed a SAH (Fisher grade 4) with IVH (Le Roux grade 2) as well as hydrocephalus and absent basal cisterns. A cerebral angiography could not detect any bleeding source. Heparin was withdrawn, and seven hours later an EVD placement was performed. The pre-operative coagulation parameters were: platelet count 39, APTT 26, ACT 134 and INR 1.1. Intra-operatively, no significant bleeding events occurred. Post-operatively, the ECMO system continued to run without heparin. ICP was further managed through hyperosmolar therapy, hyperventilation and heavy sedation (Pentobarbital coma monitored with burst suppression on continuous electroencephalography (EEG)). In addition, Nimodipine was administered to prevent cerebral vasospasm, as well as prophylactic Levetirecetam to prevent potential epileptic seizures. On ED 10, the EVD clotted and a new EVD was placed without any complications. The following day RVF developed and the patient was converted to VA ECMO. On ED 13, her pupils were of normal size and sedation was reduced, revealing signs of consciousness and movement of the left hand. Due to clotting, a week later the ECMO circuit was replaced, and the heparin infusion was restarted. On ED 30 a CT scan showed no rebleeding, ischemia, edema or hydrocephalus and the EVD was removed. Nevertheless, the patient was still GCS 3 and respiratory function continued to decline, which resulted in increased hypercapnia. Further ECMO treatment was deemed futile and, consequently, treatment was withdrawn and the patient passed away.

#### Case 5

A 67-year old man, with a history of alcohol addiction and rheumatoid arthritis, was admitted to his local hospital with a respiratory tract infection. Despite treatment with antibiotics, the patient developed septic shock with refractory respiratory failure. ECMO was indicated and cannulation for VV ECMO initiated. However, during cannulation the patient suffered multiple transient cardiac arrests and VA ECMO was commenced instead. On ED 3 a routine cranial CT scan showed no abnormalities. By ED 6, the patient’s native lung function had improved to a degree that the sedation was reduced, with the patient subsequently decannulated and withdrawn from ECMO. However, the patient did not wake up and a CT scan showed a cerebellar IPH (10 mL, largest diameter 25 mm), several minimal parietal IPHs as well as hydrocephalus and imminent cerebral herniation. Two hours later the patient underwent emergency EVD placement and evacuation of the cerebellar hematoma. Because enough time had passed since decannulation (24 hours), pre-operative hemostatic intervention was deemed unnecessary. The pre-operative coagulation parameters were: platelet count 77, APTT 30, ACT N/A, INR 1.2. Intra-operatively no significant bleeding occurred. Post-operatively, the patient was taken back to the ICU where ICP was further managed through hyperosmolar therapy and hyperventilation. After three days the EVD was removed. However, while the cranial CT scan showed resorption of IPHs, his GCS was still 3. An EEG showed slow wave activity indicative of an unspecific cerebral pathology, but a magnetic resonance imaging scan and lumbar puncture revealed no signs of encephalopathy. After transfer to the neurological ward the patient began to show signs of minor neurological improvement, but a few weeks later the patient suffered a massive pulmonary embolism. Showing no signs of further neurological improvement, life-sustaining treatment was withdrawn and the patient passed away.

Table 4 summarizes the clinical course of these five patients.

**Table 4:** Patients who were surgically treated for an ICH developed during ECMO treatment.

Variable	Case 1	Case 2	Case 3	Case 4	Case 5
Age (years)	20	35	68	47	67
Gender	Female	Female	Female	Female	Male
ECMO mode ^a^	VA	VA	VA	VV	VA
ICH classification	Frontal IPH (6.75 ml) SDH	Cerebellar IPH (23.31 mL) SAH (Fisher grade 4) IVH (Le Roux grade 4)	SAH (Fisher grade 4) IVH (Le Roux grade 4)	SAH (Fisher grade 4) IVH (Le Roux grade 2)	Cerebellar IPH (10 mL)
Radiographic features	Midline shift	Absent basal cisterns	Absent basal cisterns Hydrocephalus	Absent basal cisterns Hydrocephalus	Brain herniation Hydrocephalus
Surgical intervention	Subdural hematoma evacuation	Cerebellar hematoma evacuation	EVD placement	EVD placement	Cerebellar hematoma evacuation and EVD placement ^b^
Time from diagnosis to surgery (hours)	0.5	1	4	7	24
Pre-operative coagulation parameters	APTT 55 / ACT 204 / INR 1.0 / Platelet count 69	APTT 50 / ACT 200 / INR 1.0 / Platelet count 87	APTT 33 / ACT 153 / INR 1.1 / Platelet count 118	APTT 26 / ACT 134 / INR 1.1 / Platelet count 39	APTT 30 / ACT N/A / INR 1.2 / Platelet count 77
Intra-operative bleeding complications	No	Yes	No	No	No
Post-operative bleeding complications	Yes	N/A	No	No	No
30-day mortality	Alive	Deceased	Deceased	Deceased	Alive
6-month GOS	5	N/A	N/A	N/A	1

Abbreviations: ICH = Intracranial hemorrhage, ECMO = Extracorporeal membrane oxygenation, VA = Venoarterial, VV = Venovenous, IPH = Intraparenchymal hemorrhage, SAH = Subarachnoid hemorrhage, SDH = Subdural hemorrhage, IVH = Intraventricular hemorrhage, EVD = External ventricular drain, ACT = Activated clotting time, APTT = Activated partial thromboplastin time, INR = International normalized ratio, GOS = Glasgow outcome scale

^a^ ECMO mode at ICH diagnosis

^b^ Surgery carried out after ECMO decannulation

## Discussion

In this large retrospective cohort study, we explored predictors of poor outcome and possible management strategies following ICH development during ECMO treatment.

We found that low level of consciousness at ICH diagnosis, pre-admission antithrombotic therapy, as well as hematoma volume and secondary ICH complications (see Table 2) were significantly associated with 30-day mortality, similar to that of non-ECMO ICH patients [23–25]. Treating an ICH during ECMO treatment represents an intricate balance between pro- and anticoagulatory demands. In life-threatening situations, neurosurgical intervention may be indicated, as shown by the fact that one surgical patient made a complete recovery. This is the first cohort analysis of adult patients who developed ICH during ECMO, providing new findings that are important for future patient management and study design.

### Predictors of mortality

We found that the 30-day mortality following ICH diagnosis was 74%, which is in accordance with previous studies [7–9,26]. Out of the 17 survivors, 59% (n = 10) showed a favorable neurological outcome (GOS 4–5) after six months, indicating that there was a chance of survival with minor neurological deficits. Furthermore, low level of consciousness at ICH diagnosis, IPH, IPH volume, IVH, SAH Fisher grade, hydrocephalus, midline shift and absent basal cisterns was associated with increased 30-day mortality. These parameters are already used to predict mortality in non-ECMO ICH patients [12,24,27,28]. However, prior to this study they had not been established in the adult ECMO population.

In a recent study at our ECMO center, pre-admission antithrombotic therapy was identified as an independent predictor of ICH development during ECMO treatment [10]. Now, we also found that all eight patients with pre-admission antithrombotic therapy passed away within 30 days of ICH diagnosis. This was mainly due to the tendency of these patients to develop larger volume IPHs; 88% (n = 7) of these patients developed an IPH (with a median volume 56.70 mL) as compared to 72% (n = 41) (with a median volume of 23.31 mL) in the remaining cohort. The increased coagulopathy could be caused by tissue factor release from the damaged brain and subsequent consumption of coagulation factors [29]. Alternatively, it might be a result of reduction in the activity of coagulation factors (direct factor Xa or thrombin inhibitors), platelets (antiplatelet agents) or inhibition of their synthesis (vitamin K antagonists) [29,30]. However, we have not found any studies in the ECMO-specific population to further guide us in this area. A limitation to the variable was that we did not differentiate between different forms of antithrombotics. This could be important as they have different mechanisms of action, although differentiating and subgrouping the antithrombotics would yield extremely small subgroups and make statistical modeling difficult. For descriptive purposes, a compilation of the different forms of pre-admission antithrombotic therapy is available as a supplementary file (S2 Table). Thus, patients with pre-admission antithrombotic therapy were at increased risk of both developing an ICH and dying due to it, but its specific role needs to be further evaluated.

Reliable neurological assessment during ECMO can be difficult since patients are often sedated. Because of this, we have adopted a policy of performing routine cranial CT scans whenever the patient is referred for a thoracic or abdominal CT scan, even if there is no obvious neurological indication. Studies from centers who have adopted similar routines have shown a higher proportion of patients diagnosed with ICH [10,31,32], suggesting that low utilization of neuroimaging could contribute to underreporting of this complication. In our study, 28% (n = 18) of the ICH-cohort was diagnosed without any cranial CT indication present. Furthermore, 50% (n = 9) of these asymptomatic patients received treatment for their ICH, compared to 36% (n = 17) in the symptomatic cohort, suggesting that these ICHs were diagnosed before they had progressed into an “un-treatable” state. However, we found no significant association between the presence of neurological symptoms and mortality. Thus, asymptomatic ICH patients were more likely to undergo ICH treatment but it did not significantly affect overall risk of mortality.

Finally, we found no significant difference in mortality between VA and VV ECMO patients that developed an ICH. It has been previously hypothesized that VA ECMO patients are more inclined to develop ICH, based on an increased risk of secondary systemic thromboembolism [7,33], even though a comparison between modalities of the frequency of systemic thromboembolism has not been performed. Furthermore, in two recent retrospective studies no significant difference in ICH occurrence between VA and VV ECMO patients was found [9,10]. Thus, we have not found any difference in ICH mortality nor ICH occurrence between adult VA and VV ECMO patients, a conclusion that is supported by the available literature.

### ICH management

When an ICH occurs during ECMO treatment, the physician must balance the risk of hemorrhagic progression against that of ECMO circuit clotting and the ensuing secondary embolic events. When it came to management strategies used in our cohort, we generally found that the patients could be divided into three groups.

1. The first group was comprised of patients whose life-sustaining ECMO treatment was withdrawn following ICH diagnosis. These patients accounted for 42% (n = 27) of the entire cohort. They typically had massive IPHs, often with progressing secondary brain injury, and had showed little or no signs of native cardiorespiratory improvement during their ECMO treatment. The 30-day mortality for this group was 100%, with 96% of deaths attributed to an ICH.
2. In the second group (18% of our cohort, n = 12), the ICH was deemed to be of minimal clinical significance and no active intervention was initiated. Typically, these patients had minimal ICHs, no secondary brain injury, and showed signs of native cardiorespiratory recovery. Consequently, it was determined that treating their ICHs would do more harm than good (e.g. the increased risk of ECMO pump failure or systemic thromboembolism), even though the risk of hemorrhage progression was present. The 30-day mortality for this group was 50%, and there were no deaths attributed to an ICH (the causes of death were septic chock (n = 2), respiratory failure (n = 3) and heart failure (n = 1)). This suggests that there was no undertreatment of the ICHs.
3. The third group (40% of our cohort, n = 26) consisted of patients whose ICH diagnosis resulted in intervention. The 30-day mortality for this group was 58%, with 81% of the deaths attributed to an ICH, and the specific interventions varied depending on the situation. Whenever feasible, the patient was first decannulated—enabling intervention without increasing the risk of systemic thromboembolism or other related complications. However, since this required adequate native cardiorespiratory function, the amount of patients where this could be done was limited to nine patients. For most patients (n = 17), ICH intervention was performed during ECMO treatment, highlighting the hemostatic treatment paradox previously described. The most common forms of hemostatic intervention were heparin withdrawal and admission of platelets and anti-fibrinolytics. The most common forms of unmonitored ICP-intervention were hyperosmolar therapy and hyperventilation. Considering the risks associated with treating an ICP without monitoring, future evaluation of non-invasive ICP methods [34] should be indicated in ECMO patients when invasive ICP-monitors are contraindicated. Surgery was performed on five patients, as described in the Case series.

The surgical case series included one case of subdural hematoma evacuation, two cases of cerebellar hematoma evacuation and two cases of isolated EVD placement. In one patient (Case 5), decannulation was performed prior to surgery and pre-operative hemostatic intervention was considered unnecessary. In the remaining cases, hemostatic intervention was performed prior to surgery to decrease the risk of bleeding complications. Naturally, the time devoted to neutralizing coagulation depended on the gravity of the patient’s condition. The shortest time between heparin withdrawal and surgery was 30 minutes (Case 1), evident by the fact that this case had the highest pre-operative ACT/APTT and was the only case where extensive post-operative bleeding occurred. In addition to this case, no intra- or post-operative bleeding complications occurred during isolated EVD-placement. However, ICP catheter placement in one patient resulted in significant postoperative bleeding (Case 1). Lastly, similar to previous cases, IPH hematoma evacuation during ECMO (Case 2) resulted in extensive intra-operative bleeding and poor outcome. Generally, the surgical cases had a 30-day mortality of 60%, which was comparable to non-surgical patients. One patient (Case 1) made a full post-operative neurological recovery, making this the first described case of full return to daily life following hematoma evacuation of an ICH during ECMO treatment. While recommendations based on a handful of patients are ill advised, these cases clearly demonstrate the problems associated with the management of an ICH during ECMO treatment. Neurosurgical intervention in patients with ongoing anticoagulation is extremely hazardous. However, it may be indicated in well-selected patients where no other possible management strategies are available and the risk of death is imminent.

### Limitations

This is a retrospective study, with its inherent limitations. Probably the most important limitation was treatment bias; as extensive treatment measures were employed in some patients, while others were deemed to be beyond salvation. From a clinical standpoint these were probably correct decisions. However, it is conceivable that some patients could have survived if more extensive therapeutic strategies had been employed, which in turn infers bias in the statistical analysis. However, almost half of our patients received some form of active intervention, which seemingly escalated until futility, suggesting that treatment bias in this study was limited and that the cohort is a good example of the clinical reality.

## Conclusions

Low level of consciousness at the time of ICH diagnosis, preadmission antithrombotic therapy and more extensive hematoma components as well as related secondary complications were associated with higher 30-day mortality in ECMO patients with ICH, and should be considered when assessing these patients. Neurologically intact survival following ICH development during ECMO is achievable. Treating an ICH during ECMO represents an intricate balance between pro- and anticoagulatory demands. Furthermore, neurosurgery may be advocated in life-threatening lesions, as illustrated by our case series.

## Supporting information

S1 Dataset All raw data used in the study. (XLSX)

S1 Table Mean clinical and laboratory values, between ICH diagnosis and decannulation/death, in patients who continued on ECMO support. (DOCX)

S2 Table Detailed pre-admission antithrombotic therapy. (DOCX)
