# Supplementary material for: Management of intracranial hemorrhage in adult patients on extracorporeal membrane oxygenation (ECMO): An observational cohort study
Source: PLoS One. 2017 Dec 21;12(12):e0190365. doi: 10.1371/journal.pone.0190365 (PMC5739492; doi:10.1371/journal.pone.0190365)
Supplement: S1 Table — (DOCX) [file pone.0190365.s002.docx]

**S1 Table. Mean clinical and laboratory values, between ICH diagnosis and decannulation/death, in patients who continued on ECMO support.**

| **Variable** | **Alive (n=17)** | **Deceased (n=21)** | **p-value** |
| --- | --- | --- | --- |
| MAP (mmHg) | 69 (66–74) (4 missing, 24%) | 69 (66.5–76) | >0.05 |
| ACT (sec.) | 189 (180–204.5) (4 missing, 24%) | 193 (168–218.50) | >0.05 |
| APTT (sec.) | 62.50 (48–72.75) (3 missing, 18%) | 68.50 (42.25–82.25) (1 missing, 5%) | >0.05 |
| INR | 1.15 (1.08–1.35) (3 missing, 18%) | 1.20 (1.00–1.30) (2 missing, 10%) | >0.05 |
| Platelet count (x10^9^/mL) | 58 (35.75–124.75) (3 missing, 18%) | 64.50 (41.50–80.75) (1 missing, 5%) | >0.05 |
| Fibrinogen (g/L) | 3.35 (2.80–5.78) (3 missing, 18%) | 3.45 (2.43–4.85) (1 missing, 5%) | >0.05 |
| PvCO_2_ (kPa) | 5.40 (4.85–6.10) (4 missing, 24%) | 5.50 (5.23–6.07) (1 missing, 5%) | >0.05 |

Abbreviations: MAP= Mean arterial pressure, ACT= Activated clotting time, APTT= Activated partial thromboplastin time, INR= International normalized ratio

Values are expressed as median (interquartile range)
