# Supplementary material for: Management of intracranial hemorrhage in adult patients on extracorporeal membrane oxygenation (ECMO): An observational cohort study
Source: PLoS One. 2017 Dec 21;12(12):e0190365. doi: 10.1371/journal.pone.0190365 (PMC5739492; doi:10.1371/journal.pone.0190365)
Supplement: S2 Table — (DOCX) [file pone.0190365.s003.docx]

**S2 Table. Detailed pre-admission antithrombotic therapy.**

| **Antithrombotic therapy** | **Patients (n)** |
| --- | --- |
| Warfarin | 3 |
| Heparin | 1 |
| ASA | 2 |
| ASA + Clopidogrel | 1 |
| ASA + Tinzaparin | 1 |

Abbreviations: ASA = Acetylsalicylic acid
